# Novel deep learning model for more accurate prediction of drug-drug interaction effects

**DOI:** 10.1186/s12859-019-3013-0

**Published:** 2019-08-06

**Authors:** Geonhee Lee, Chihyun Park, Jaegyoon Ahn

**Affiliations:** 1 Department of Computer Science and Engineering, Incheon National University, Incheon, 22012 South Korea; 2 Department of Computer Sciences, Yonsei University, Seoul, 03722 South Korea; 3 Department of Computer Science and Engineering, Incheon National University, Incheon, 22012 South Korea

**Keywords:** Drug-drug interaction, Deep learning, Autoencoder, Similarity profile

## Abstract

**Background:**

Predicting the effect of drug-drug interactions (DDIs) precisely is important for safer and more effective drug co-prescription. Many computational approaches to predict the effect of DDIs have been proposed, with the aim of reducing the effort of identifying these interactions in vivo or in vitro, but room remains for improvement in prediction performance.

**Results:**

In this study, we propose a novel deep learning model to predict the effect of DDIs more accurately.. The proposed model uses autoencoders and a deep feed-forward network that are trained using the structural similarity profiles (SSP), Gene Ontology (GO) term similarity profiles (GSP), and target gene similarity profiles (TSP) of known drug pairs to predict the pharmacological effects of DDIs. The results show that GSP and TSP increase the prediction accuracy when using SSP alone, and the autoencoder is more effective than PCA for reducing the dimensions of each profile. Our model showed better performance than the existing methods, and identified a number of novel DDIs that are supported by medical databases or existing research.

**Conclusions:**

We present a novel deep learning model for more accurate prediction of DDIs and their effects, which may assist in future research to discover novel DDIs and their pharmacological effects.

**Electronic supplementary material:**

The online version of this article (10.1186/s12859-019-3013-0) contains supplementary material, which is available to authorized users.

## Background

Combination drug therapies are becoming a promising approach for several diseases including cancer, hypertension, asthma and AIDS, since they can increase drug efficacy, decrease drug toxicity or reduce drug resistance [1]. However, the combination of drugs may result in interactions between drugs (drug-drug interactions, DDIs), which are a major cause of adverse drug events (ADEs) [2, 3]. It is estimated that DDIs are associated with 30% of all reported ADEs [4]. In addition, ADEs due to critical DDIs have led to the withdrawal of drugs from the market [5]. Therefore, precise prediction of the effect of DDIs is important for safer and improved prescription to patients.

DDIs can be identified with in vivo models using high-throughput screening [6]. However, the price of such procedures is relatively high, and testing large numbers of drug combinations is not practical [7]. To reduce the number of possible drug combinations, numerous computational approaches have been proposed [8–15].

In some of these computational approaches, drug-target networks are constructed, and DDIs are detected by measuring the strength of network connections [13], or by identifying drug pairs that share drug targets or drug pathways using the random walk algorithm [14].

Other major categories of these computational approaches are based on the structural and side effect similarities of drug pairs. For example, Gottlieb et al. proposed the Inferring Drug Interactions (INDI) method, which predicts novel DDIs from chemical and side effect similarities of known DDIs [8], and Vilar et al. used similarities of fingerprints, target genes, and side effects of drug pairs [9, 10]. Cheng et al. constructed features from Simplified Molecular-Input Line-Entry System (SMILES) data and side effect similarity of drug pairs, and applied support vector machines to predict DDIs [11]. Zhang et al. constructed a network of drugs based on structural and side effect similarities, and applied a label propagation algorithm to identify DDIs [12]. Recently, Ryu et al. proposed DeepDDI, a computational framework that calculates structural similarity profiles (SSP) of DDIs, reduces features using principal component analysis (PCA), and feeds them to the feed-forward deep neural network [15]. The platform generated 86 labeled pharmacological DDI effects, so DeepDDI is basically a multi-classification (multi-label classification) model.

To increase the classification accuracy in the present study, we proposed a novel deep learning based model that uses additional features from target genes and their known functions. We constructed target similarity profiles (TSP) and Gene Ontology (GO) term similarity profiles (GSP), as well as SSP. Because the input size is too large when combining TSP, GSP, and SSP, we used an autoencoder [16] to reduce the feature. Our autoencoder model is trained to minimize the difference between input and output, and at the same time, trained to minimize the error of prediction of DDI labels. Our model showed improved classification accuracy, and we were able to identify novel DDIs with their pharmacological effects.

## Results

**Fig. 1:**
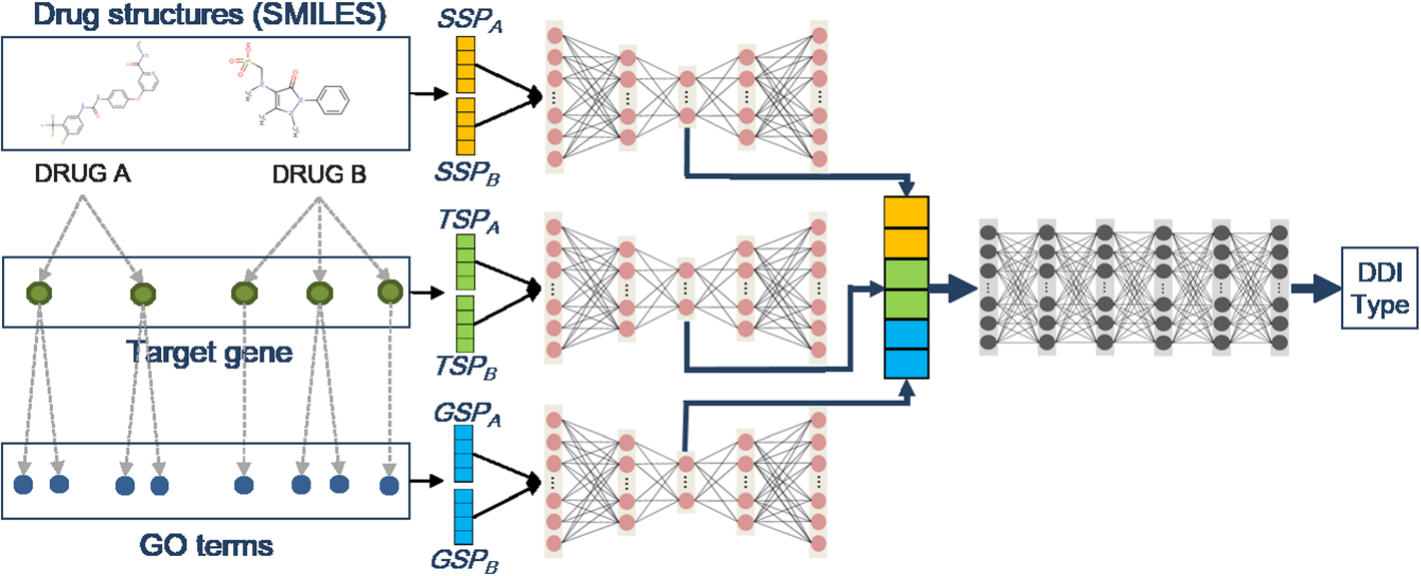
Overview of the prediction model

We developed a novel deep learning model to predict pharmacological effects of DDIs. This model uses an autoencoder to reduce the dimensions of three similarity profiles of drug pairs, and uses a deep feed-forward network that predicts DDI type from reduced similarity profiles. Three similarity profiles are calculated using the chemical structures (SSP), target genes (TSP), and target genes’ biological/molecular function (GSP) of known drug pairs. The entire process is depicted in Fig. 1, and detailed descriptions are provided in the methods section.

To train our model, we downloaded 396,454 known DDIs of 177 types, and SMILES and target gene information for drugs from DrugBank [17]. Functional Interaction (FI) networks were downloaded from BioGrid [18]. FI networks are composed of 22,032 genes. The GO database was downloaded from the Gene Ontology Consortium [19, 20]. The GO database is composed of 45,106 GO terms, and we used 29,692 GO terms in biological processes. Drugs with no target gene information were excluded, and DDI types with fewer than five DDIs were excluded. Finally, 188,258 DDIs of 106 types (Additional file 1: Table S1) and 1597 drugs were used for the experiments.

Our model was learned using different combinations of SSP, TSP, and GSP. The accuracy, macro precision, macro recall, micro precision, micro recall, and the area under the Precision/Recall curve (AUPRC) were calculated using 5-fold cross-validation. These performance metrics are as follows:

$$ \mathrm{Accuracy}=\frac{1}{n}\sum \limits_{i=1}^n{x}_i=\left\{\ \begin{array}{c}1\  if\ {y}_i\ge 0.5\\ {}\ 0\  otherwise\ \end{array}\right. $$

$$ \mathrm{Macro}\ \mathrm{recall}=\frac{1}{l}\sum \limits_{i=1}^l\frac{TP_i}{TP_i+{FN}_i} $$

$$ \mathrm{Macro}\ \mathrm{precision}=\frac{1}{l}\sum \limits_{i=1}^l\frac{TP_i}{TP_i+{FP}_i} $$

$$ \mathrm{Micro}\ \mathrm{recall}=\frac{\sum_{i=1}^l{TP}_i}{\sum_{i=1}^l{TP}_i+{FN}_i} $$

$$ \mathrm{Micro}\ \mathrm{precision}=\frac{\sum_{i=1}^l{TP}_i}{\sum_{i=1}^l{TP}_i+{FP}_i} $$

where *n* and *l* indicate number of samples and DDI types respectively, *y*_*i*_ is a predicted value of true DDI type in the DrugBank database of sample *i*, and *TP, TN, FP* and *FN* are true positive, true negative, false positive and false negative, respectively.

**Fig. 2:**
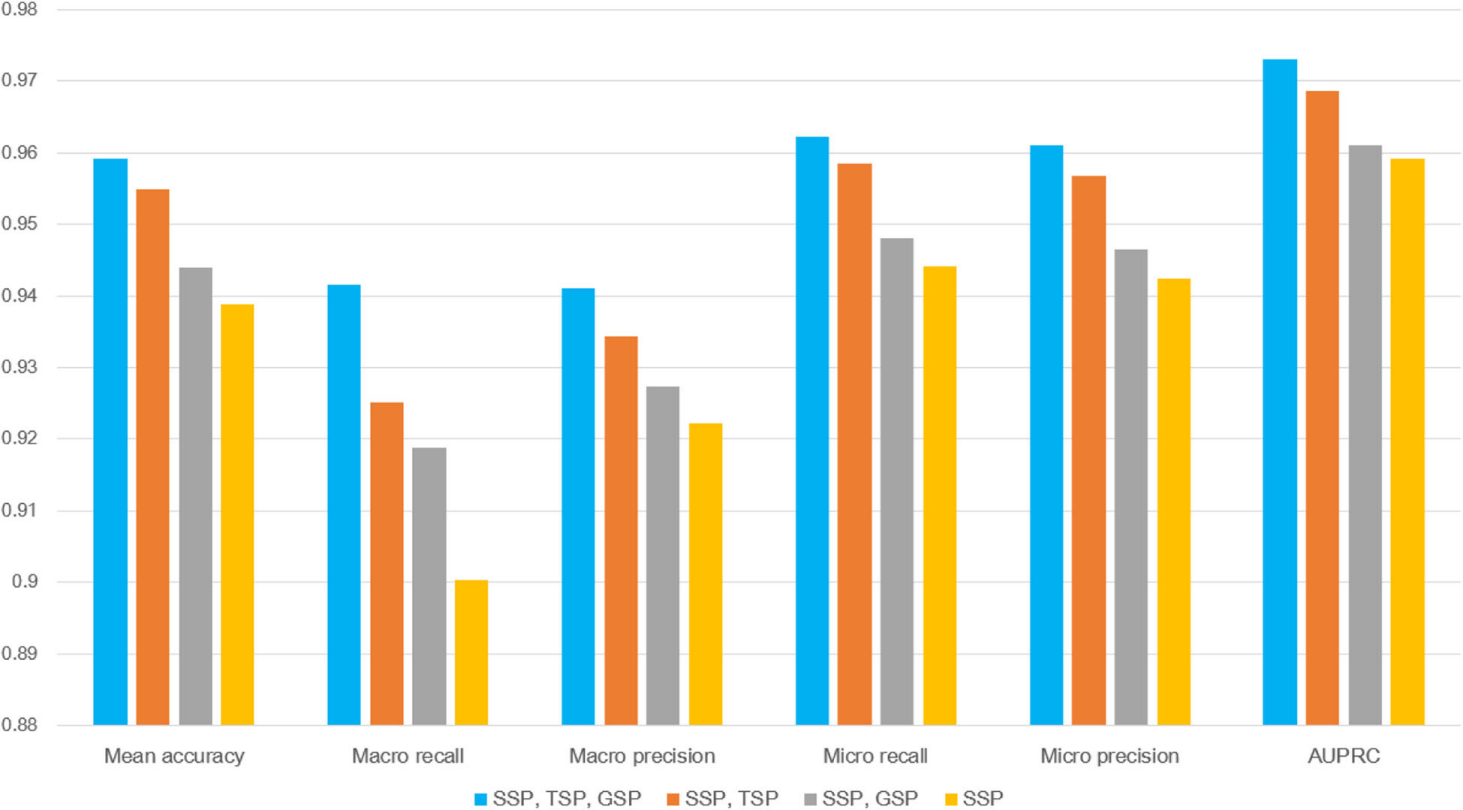
Comparison with different data combinations

**Fig. 3:**
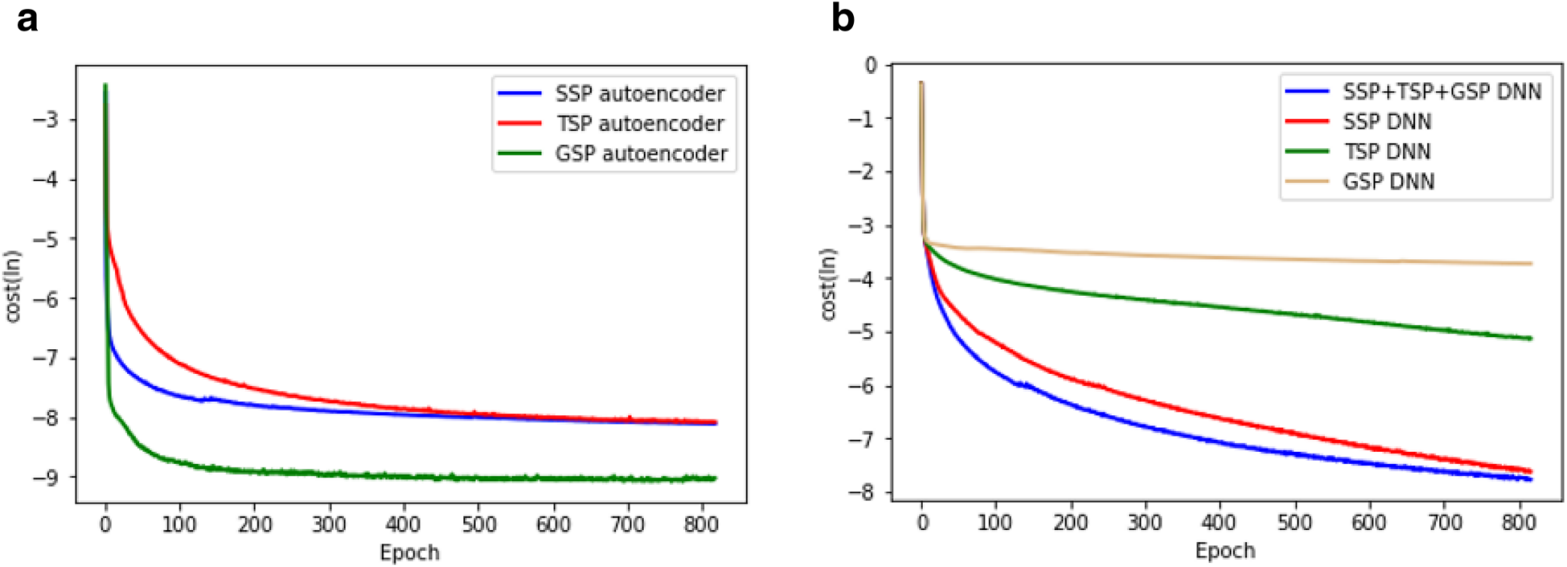
Cost curve of **a** different autoencoders and **b** deep feed-forward neural networks for different similarity profiles

Figure 2 shows that incorporating TSP and GSP increases the classification accuracy. The tests using GSP and TSP only, and those using both GSP and TSP, did not generate good classification accuracy (< 0.5). We were also able to observe that TSP and GSP increase classification accuracy in terms of AUPRC. Figure 3 shows cost curves for an autoencoder and deep feed-forward networks, and it can be observed that while the deep feed-forward networks for TSP and GSP converge, the costs are relatively large. Although GSP and TSP are not good single similarity measures, they increased the prediction performance using SSP.

**Fig. 4:**
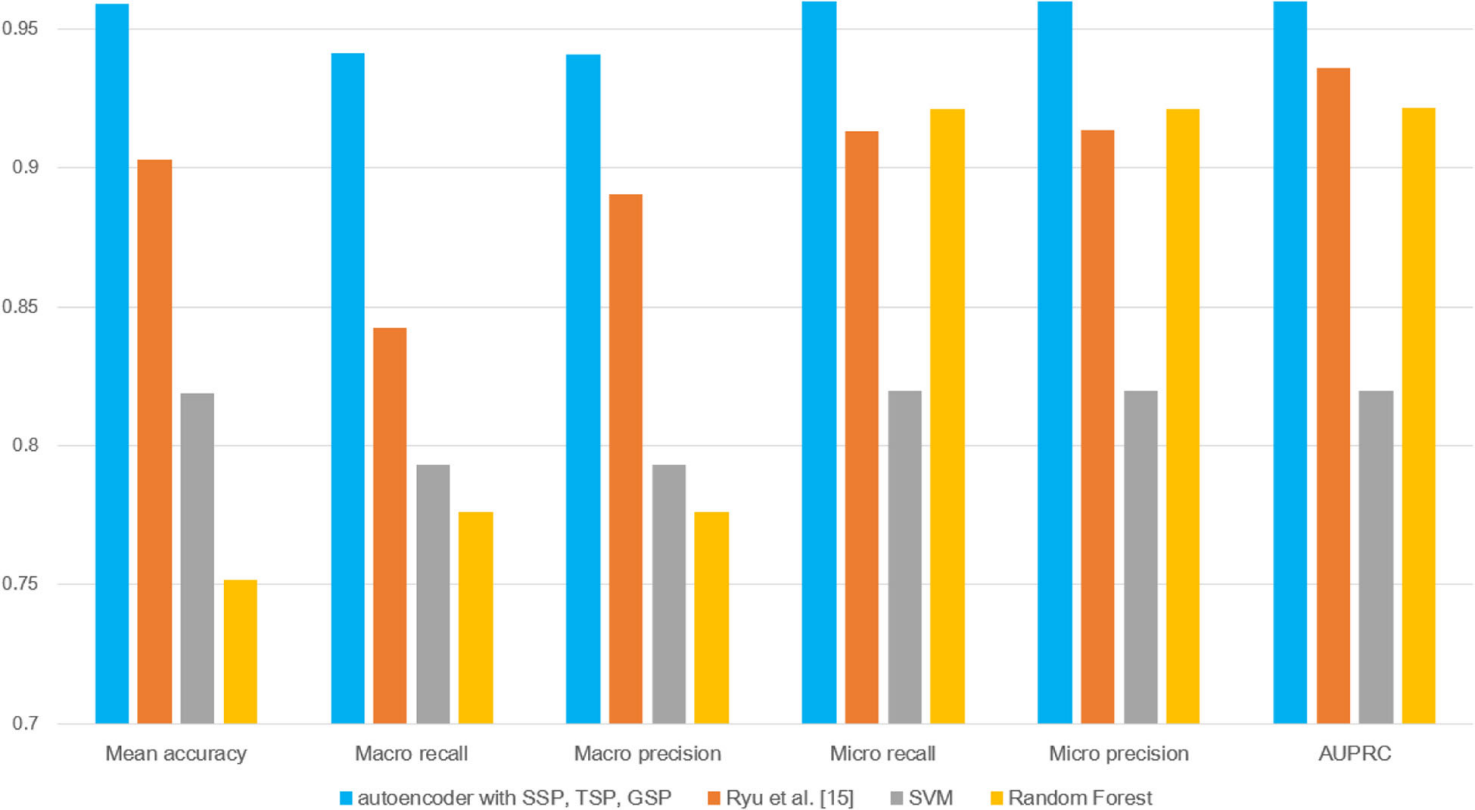
Comparison with different machine learning models

**Fig. 5:**
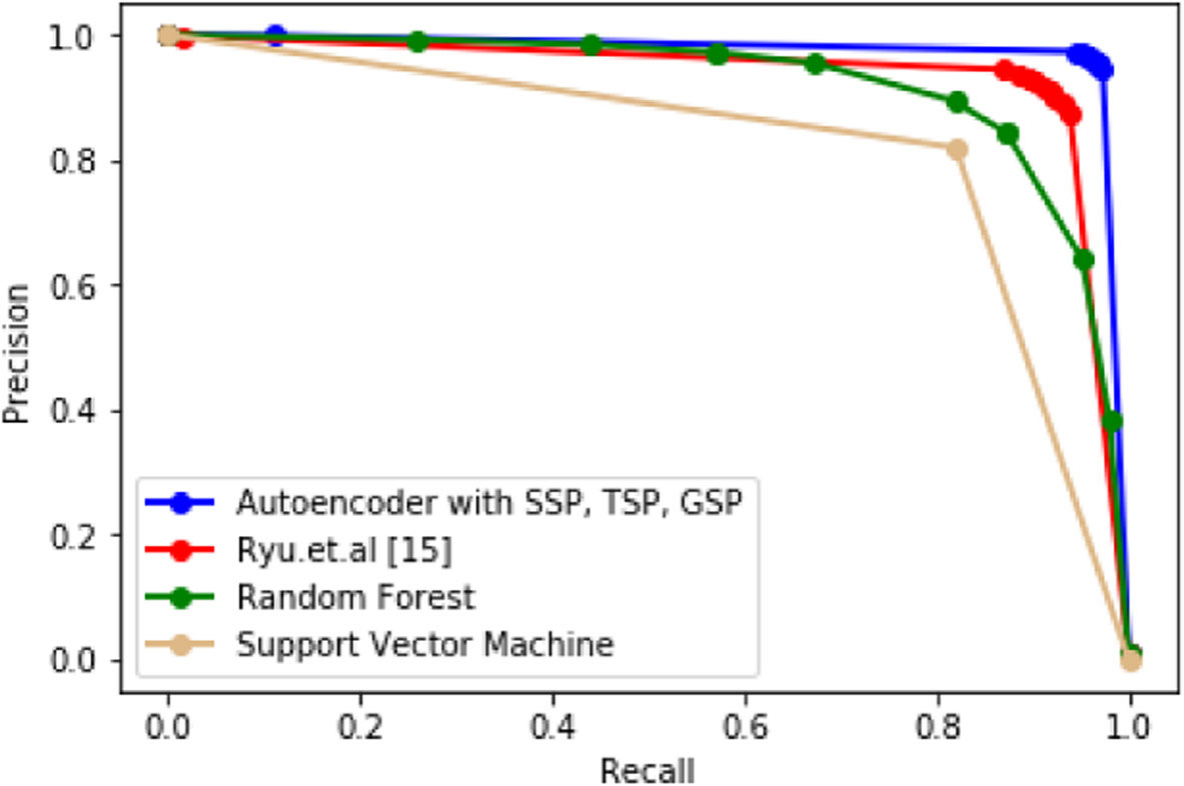
Precision/Recall curves of machine learning models

We can see that SSP using the autoencoder (yellow in Fig. 2) generates superior results to those of SSP using PCA [15] in Figs. 4 and 5. We can also confirm that the proposed model shows better performance than baseline methods such as SVM or Random Forest. The hyper-parameters for SVM and Random Forest are provided in Table 1. For the proposed model and that of Ryu et al. [15] in Figs. 2, 4, and 5, the number of features was reduced to 200 using the autoencoder or PCA, and the features for SVM and Random Forest were not reduced.

**Table 1:** Hyper-parameters of Random Forest and SVM

Random Forest				SVM			
Criterion	Minimum samples leaf	Minimum samples split	Number of estimators	C	Loss	Maximum iteration	Penalty
Gini impurity	1	2	10	1	Square of the hinge loss	1000	L2

**Fig. 6:**
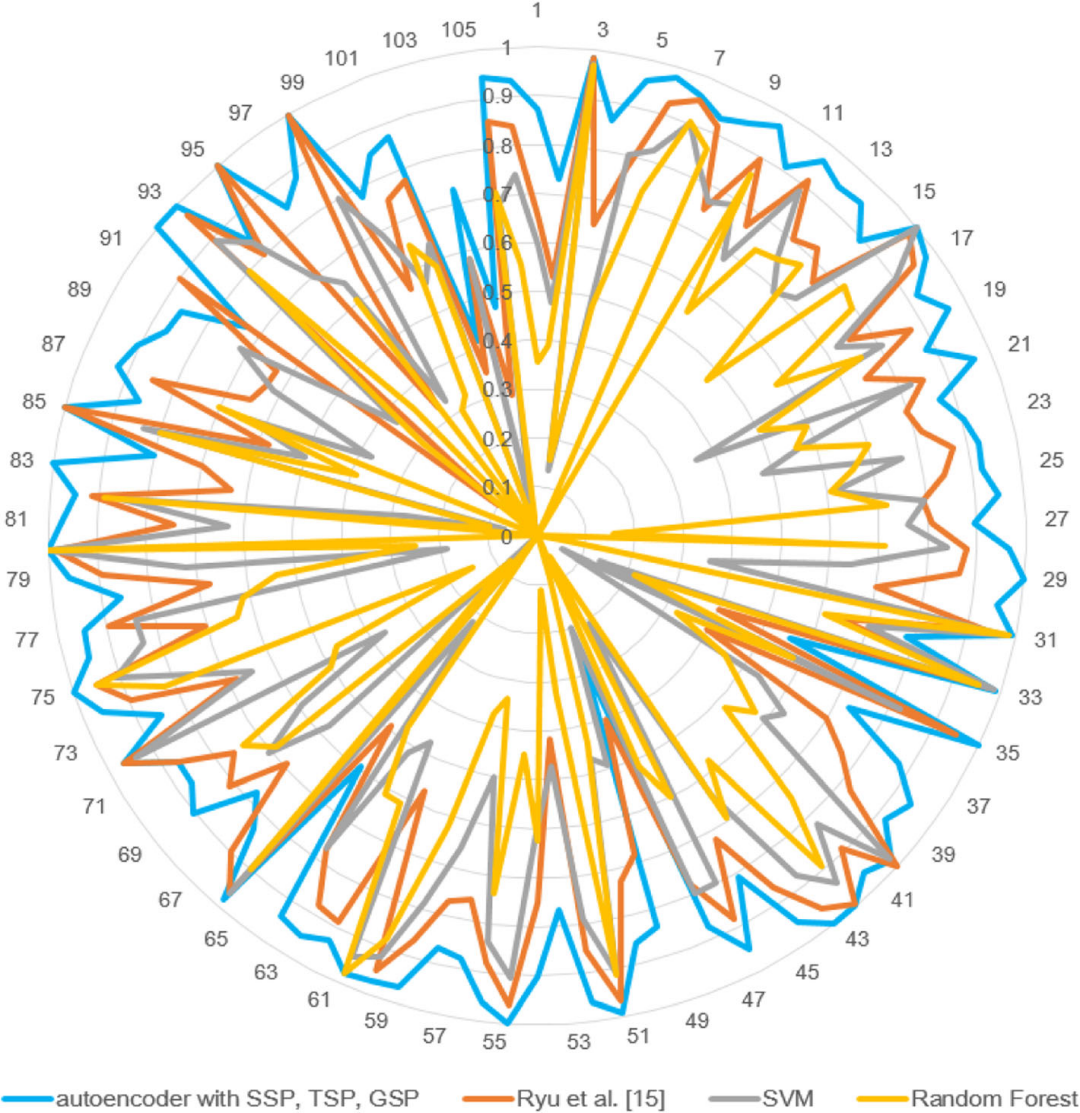
Accuracies of methods for each DDI types

**Fig. 7:**
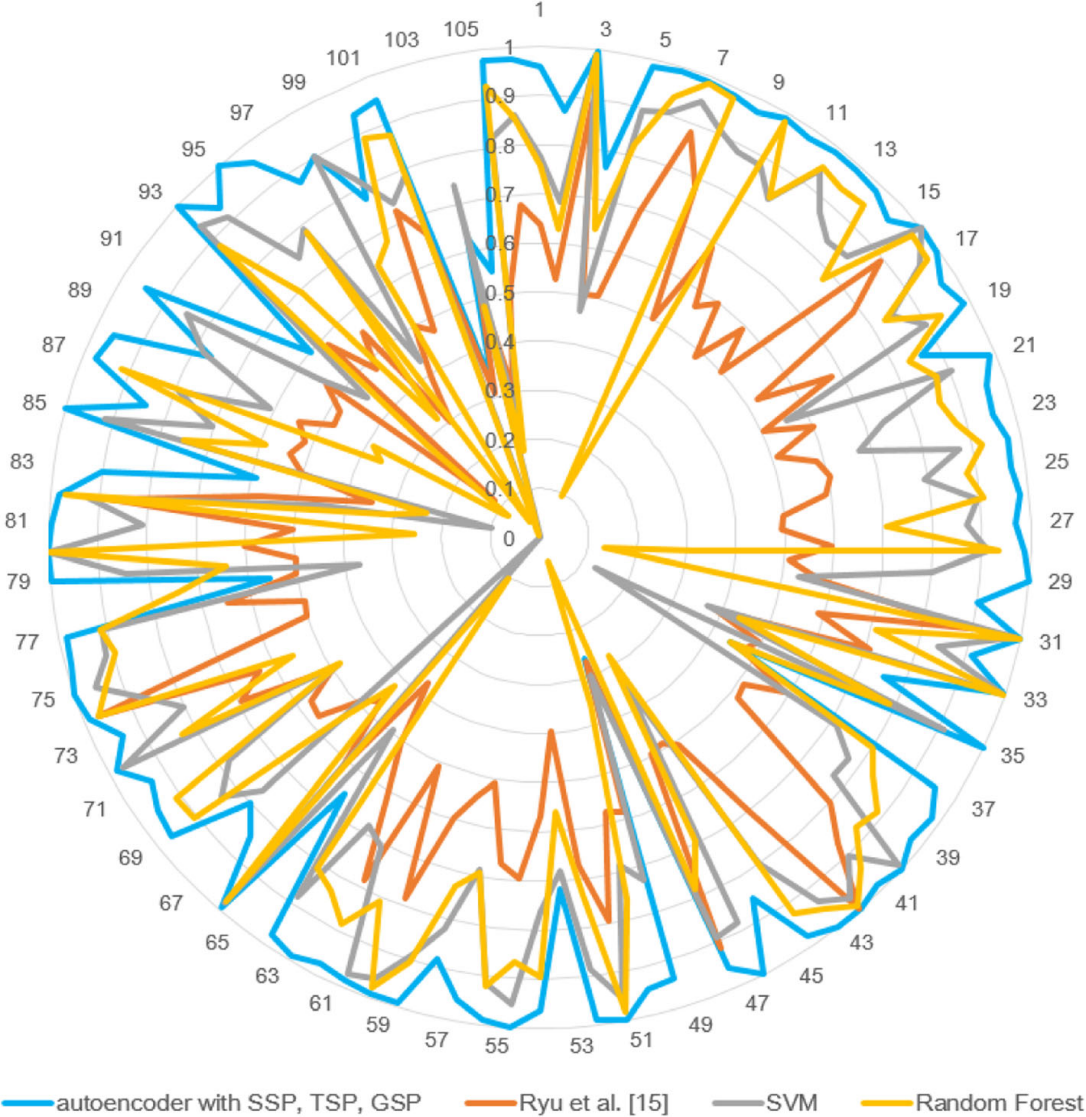
AUPRC of methods for each DDI types

To observe the performance of each method more specifically, we compared the results for each DDI type. Greater or the same classification accuracy was observed for 101 out of 106 DDI types in two cases using the proposed model (Figs. 6 and 7).

## Discussions

Among the true positive predictions in the 5-fold cross-validation results, we selected drug pairs with a predicted value of other DDI type (not the ground truth from Drugbank v5.1.1) greater than or equal to 0.5, and provided these in Additional file 1: Table S2. Among 580 such drug pairs, 86 (14.8%) drug pairs were supported by other databases or existing studies. Among the 86 drug pairs that were supported, we show 12 drug pairs with prediction score > 0.8 in Table 2. The types of the first three DDIs in Table 2 were 100, 100, and 76 in DrugBank v5.1.1, but they were updated to 86, 86, and 18 in DrugBank v5.1.2, and our prediction scores were very high for these three DDIs.

**Table 2:** Predicted DDI types of drug pairs

Drug A	Drug B	DDI type (Drugbank v5.1.1)^a^	Score (Drugbank v5.1.1)	DDI type (new prediction)^a^	Score (new prediction)	Reference
Amodiaquine	Pyrimethamine	100	0.999999762	86	0.999997854	DrugBank v 5.1.2
Amodiaquine	Cholecalciferol	100	0.99999392	86	0.996211529	DrugBank v 5.1.2
Betrixaban	Rivaroxaban	76	0.518932223	18	0.989234567	DrugBank v 5.1.2
Disopyramide	Asenapine	24	0.775597036	72	0.984019518	https://www.pdr.net
Nefazodone	Amoxapine	19	0.581683695	56	0.907319307	https://online.epocrates.com
Ulipristal	Pentobarbital	99	0.999979138	104	0.902777851	https://www.pdr.net
Fingolimod	Dronedarone	22	0.547105849	14	0.881702363	[21]
Trazodone	Methylene blue	56	0.697363019	96	0.879396319	https://www.pdr.net
Fluorouracil	Metronidazole	100	0.782166064	102	0.864007413	https://www.drugs.com
Tacrolimus	Escitalopram	86	0.511624634	14	0.851550758	[22, 21]
Tacrolimus	Citalopram	86	0.511636794	14	0.851546466	[22, 23]
Methadone	Escitalopram	86	0.537029743	14	0.849954724	[22, 21]

^a^ 14: DRUG_A may increase the QTc-prolonging activities of DRUG_B

18: DRUG_A may increase the anticoagulant activities of DRUG_B

19: DRUG_A may increase the antihypertensive activities of DRUG_B

22: DRUG_A may increase the arrhythmogenic activities of DRUG_B

24: DRUG_A may increase the bradycardic activities of DRUG_B

56: DRUG_A may increase the serotonergic activities of DRUG_B

72: The risk or severity of QTc prolongation can be increased when DRUG_A is combined with DRUG_B

76: The risk or severity of bleeding can be increased when DRUG_A is combined with DRUG_B

86: The risk or severity of hypotension can be increased when DRUG_A is combined with DRUG_B

96: The risk or severity of serotonin syndrome can be increased when DRUG_A is combined with DRUG_B

99: The serum concentration of DRUG_A can be decreased when it is combined with DRUG_B

100: The serum concentration of DRUG_A can be increased when it is combined with DRUG_B

102: The serum concentration of the active metabolites of DRUG_A can be increased when DRUG_A is used in combination with DRUG_B

104: The therapeutic efficacy of DRUG_A can be decreased when used in combination with DRUG_B

Our work has two potential limitations. First, DDIs in DrugBank are mostly inferred pharmacokinetic interactions, so the DDIs predicted by the proposed model, as well as their clinical consequences should be validated. Second, the optimal values for the hyper-parameters such as learning rate, number of hidden units/layers, and drop-out rate were obtained by iterative experiments for our setting, so the experimental results can be changed for different settings including different dataset version or experimental environment. We recommend that potential users of the proposed model identify their own optimal hyper-parameters through cross-validation.

## Conclusion

In this study, we propose a novel deep learning model for more accurate prediction of the pharmacological effects of DDIs. The proposed model is trained using three similarity profiles, SSP, TSP, and GSP, of each drug. Those similarity profiles are reduced using autoencoders and fed into a deep feed-forward network to predict the type of each DDI. The proposed model showed improved classification accuracy over existing models. We found that GSP and TSP can increase the prediction performance. We also predicted new effects of numerous DDIs, many of which were supported by a number of databases or previous studies.

## Methods

### Similarity measures

We used three similarity measures using three profiles, structural similarity profile (SSP), target gene similarity profile (TSP), and Gene Ontology (GO) term similarity profile (GSP).

SSP for drug *A* is a vector of structural similarity values between *A* and the rest of the drugs. A structural similarity between two drugs is a Tanimoto coefficient [24] between their binary vectors (fingerprints) converted from their SMILES [25]. SSP of drug *A* can be represented as S*SP*_*A*_ = {*SS*_*AA*_, *SS*_*AB*_, *SS*_*A*C_, …}, where *SS*_*Ax*_ is the Tanimoto coefficient between drug *A* and *X*.

TSP for drug *A* is a vector of target gene similarity values between *A* and the rest of the drugs. A target gene similarity between drugs *A* and *B* is calculated with the following formula:

$$ {TS}_{AB}=\frac{\left|\Big\{\left(x,y\right)\in {G}_A\times {G}_B\ \right|\ d\left(x,y\right)\le {t}_A\Big\}\mid }{\mid \left\{\left(x,y\right)\in {G}_A\times {G}_B\right\}\mid } $$

$$ {t}_A=\mathit{\max}\ \left\{\ d\left(x,y\right)\ \right|\ x,y\in {G}_A\Big\} $$

where *G*_*A*_ and *G*_*B*_ are target genes for drug *A* and *B*, and *d* (*x*, *y*) is a distance between genes *x* and *y* in the FI network. In short, a target gene similarity between drugs *A* and *B* is the ratio of gene pairs that have a shorter distance than the maximum distance *t*_*A*_. TSP of drug *A* can be represented as *TSP*_*A*_ = {*TS*_*AA*_, *TS*_*AB*_, *TS*_*AC*_, …}.

Calculation of GSP is the same as that of TSP, except that gene and FI network are substituted with GO term and GO graph, respectively. GSP of drug *A* can be represented as *GSP*_*A*_ = {*GS*_*AA*_, *GS*_*AB*_, *GS*_*AC*_, …}, where *GS*_*AB*_ is similar to *TS*_*AB*_. The length of SSP, TSP, and GSP of a drug is 1597, which is same as the number of all drugs.

### Model for prediction of DDI type

The model for prediction of DDI type is composed of three autoencoders and one deep feed-forward network. The autoencoders are used to reduce the dimensions of SSP, TSP, and GSP. Three autoencoders are homogeneous, and have input and output layers of which the size is 3194 (= 1597 × 2), and 3 hidden layers of which the sizes are 1000, 200, and 1000, respectively. The reduced profile pairs are concatenated and fed to the deep feed-forward network. The deep feed-forward network has an input layer of size 600; 6 hidden layers of size 2000; and an output layer of size 106, which is same as the number of DDI types.

The batch size of input is 256, and the learning rates of the autoencoder and feed-forward network are 0.001 and 0.0001, respectively. The activation functions for the autoencoder and feed-forward network are sigmoid and ReLU [26]. We used sigmoid for the activation function for the output layer of the feed-forward network. The number of epochs is 850, and we used Adam for the feed-forward network and RMSprop for the autoencoder as an optimizer [27]. To avoid overfitting, we applied dropout with a drop rate of 0.3 and batch normalization for the feed-forward network and autoencoders.

For each epoch, three autoencoders are independently trained to minimize the difference of input and output. Then the feed-forward network is trained with the reduced profile pairs as input. The training is performed to minimize the sum of costs from the three autoencoders and the feed-forward network. Therefore, the autoencoders are trained twice, and encode profiles so as to predict the DDI type more accurately.

## Additional file

Additional file 1: **Table S1.** DDI types. **Table S2.** Prediction of DDI (prediction score ≥ 0.5). (XLSX 59 kb)

## Data Availability

DrugBank, https://www.drugbank.ca/releases/latest

## Abbreviations

ADEs: Adverse drug events
DDIs: Drug-drug interactions
GO: Gene ontology
GSP: GO term similarity profiles
NSCLC: Non-small cell lung cancer
SMILES: Molecular-Input Line-Entry System
SSP: Structural similarity profiles
TSP: Target gene similarity profiles
